# 1,1′-Methyl­enedipyridinium dichloride monohydrate

**DOI:** 10.1107/S1600536810015096

**Published:** 2010-04-30

**Authors:** Wen-Zhen Fu, Wei-Juan Wang, Yun-Yin Niu, Seik Weng Ng

**Affiliations:** a Department of Chemistry, Zhengzhou University, Zhengzhou 450052, People’s Republic of China; b Department of Chemistry, University of Malaya, 50603 Kuala Lumpur, Malaysia

## Abstract

In the crystal structure of the title salt, C_11_H_12_N_2_^2+^·2Cl^−^·H_2_O, the dication adopts a butterfly shape [dihedral angle between rings = 69.0 (1)°] with the water mol­ecule lying in the V-shaped cavity. Each O—H bond of the water molecule lies parallel to an aromatic ring and forms an O—H⋯Cl inter­action to a chloride anion. The methyl­ene C atom in the dication and the water O atoms lie on special positions of twofold site symmetry.

## Related literature

For the synthesis, see: Almarzoqi *et al.* (1986 ▶). For the crystal structure of dipyridiniomethane diiodide, see: Brüdgam & Hartl (1986 ▶). For background to the use of similar compounds in the synthesis of coordination polymers, see: Niu *et al.* (2008 ▶).
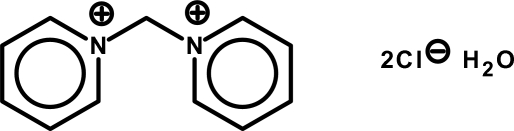


## Experimental

### 

#### Crystal data

C_11_H_12_N_2_^2+^·2Cl^−^·H_2_O
*M* *_r_* = 261.14
Orthorhombic, 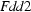

*a* = 16.3384 (15) Å
*b* = 19.0958 (18) Å
*c* = 7.7916 (7) Å
*V* = 2430.9 (4) Å^3^
*Z* = 8
Mo *K*α radiation
μ = 0.51 mm^−1^
*T* = 100 K
0.30 × 0.15 × 0.10 mm

#### Data collection

Bruker SMART APEX diffractometer
Absorption correction: multi-scan (*SADABS*; Sheldrick, 1996 ▶) *T*_min_ = 0.861, *T*_max_ = 0.950
5641 measured reflections
1389 independent reflections
1333 reflections with *I* > 2σ(*I*)
*R*_int_ = 0.028

#### Refinement

*R*[*F* ^2^ > 2σ(*F* ^2^)] = 0.022
*wR*(*F* ^2^) = 0.056
*S* = 1.04
1389 reflections
78 parameters
2 restraints
H atoms treated by a mixture of independent and constrained refinement
Δρ_max_ = 0.23 e Å^−3^
Δρ_min_ = −0.16 e Å^−3^
Absolute structure: Flack (1983 ▶), 642 Friedel pairs
Flack parameter: 0.01 (6)

### 

Data collection: *APEX2* (Bruker, 2009 ▶); cell refinement: *SAINT* (Bruker, 2009 ▶); data reduction: *SAINT*; program(s) used to solve structure: *SHELXS97* (Sheldrick, 2008 ▶); program(s) used to refine structure: *SHELXL97* (Sheldrick, 2008 ▶); molecular graphics: *X-SEED* (Barbour, 2001 ▶); software used to prepare material for publication: *publCIF* (Westrip, 2010 ▶).

## Supplementary Material

Crystal structure: contains datablocks global, I. DOI: 10.1107/S1600536810015096/jh2148sup1.cif

Structure factors: contains datablocks I. DOI: 10.1107/S1600536810015096/jh2148Isup2.hkl

Additional supplementary materials:  crystallographic information; 3D view; checkCIF report

## Figures and Tables

**Table 1:** Hydrogen-bond geometry (Å, °)

*D*—H⋯*A*	*D*—H	H⋯*A*	*D*⋯*A*	*D*—H⋯*A*
O1w—H1⋯Cl1	0.85 (1)	2.37 (1)	3.216 (1)	177 (2)

**Fig. 1.**
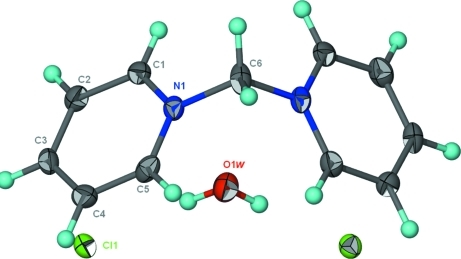
Thermal ellipsoid plot (Barbour, 2001) of [C11H12N2]2+ 2Cl-.H2O at the 50% probability level; hydrogen atoms are drawn as spheres of arbitrary radius.

## supplementary crystallographic information

### Comment

Dichloromethane reacts with tertiary amines under high pressure to form bis-ammonium salts, as exemplified by its reaction with pyridine (Almarzoqi *et al.*, 1986). This class of compounds represents a class of ammonium salts that are excellent directing regents for the construction of metal–organic architectures (Niu *et al.*, 2008). The structure of the dipyridiniomethane dichloride homolog has not been reported but the structure of the anhydrous diiodide has been known for some time. The salt shows short cation–iodine contacts [3.620 (7)–3.742 (9) Å], which are believed to render the salt useful for studing charge-transfer processes in the solid state (Brüdgam & Hartl, 1986). Dipyridiniomethane dichloride crystallizes as a dihydrate (Scheme I, Fig. 1). The dication lies about a two-fold rotation axis that passes through the methylene carbon atom [N–C–N 110.2 (2) °]; the water molecule also lies on a two-fold rotation axis; the molecule is hydrogen–bond donor to the chorine atom.

### Experimental

The compound was synthesized as described by Almarzoqi *et al.* (1986). The attempt to react it with CuI and [NH_4_]_2_[WO_2_S_2_] in a methanol-DMF mixture. returned the salt as rice-bead shaped yellow crystals.

### Refinement

Hydrogen atoms were placed in calculated positions (C—H 0.95 to 0.99 Å) and were included in the refinement in the riding model approximation, with *U*(H) set to 1.2*U*_eq_(C). The water H-atom was located in a difference Fourier map, and was refined with a restraint of O–H 0.84±0.01 Å.

### Crystal data

**Table:**

C_11_H_12_N_2_^2+^·2Cl^−^·H_2_O	*F*(000) = 1088
*M**_r_* = 261.14	*D*_x_ = 1.427 Mg m^−^^3^
Orthorhombic, *F**d**d*2	Mo *K*α radiation, λ = 0.71073 Å
Hall symbol: F2 -2d	Cell parameters from 2704 reflections
*a* = 16.3384 (15) Å	θ = 3.1–28.2°
*b* = 19.0958 (18) Å	µ = 0.51 mm^−^^1^
*c* = 7.7916 (7) Å	*T* = 100 K
*V* = 2430.9 (4) Å^3^	Bead, yellow
*Z* = 8	0.30 × 0.15 × 0.10 mm

### Data collection

**Table:**

Bruker SMART APEX diffractometer	1389 independent reflections
Radiation source: fine-focus sealed tube	1333 reflections with *I* > 2σ(*I*)
graphite	*R*_int_ = 0.028
ω scans	θ_max_ = 27.4°, θ_min_ = 3.1°
Absorption correction: multi-scan (*SADABS*; Sheldrick, 1996)	*h* = −21→21
*T*_min_ = 0.861, *T*_max_ = 0.950	*k* = −23→24
5641 measured reflections	*l* = −10→10

### Refinement

**Table:**

Refinement on *F*^2^	Secondary atom site location: difference Fourier map
Least-squares matrix: full	Hydrogen site location: inferred from neighbouring sites
*R*[*F*^2^ > 2σ(*F*^2^)] = 0.022	H atoms treated by a mixture of independent and constrained refinement
*wR*(*F*^2^) = 0.056	*w* = 1/[σ^2^(*F*_o_^2^) + (0.0329*P*)^2^ + 1.2206*P*] where *P* = (*F*_o_^2^ + 2*F*_c_^2^)/3
*S* = 1.03	(Δ/σ)_max_ = 0.001
1389 reflections	Δρ_max_ = 0.23 e Å^−^^3^
78 parameters	Δρ_min_ = −0.16 e Å^−^^3^
2 restraints	Absolute structure: Flack (1983), 642 Friedel pairs
Primary atom site location: structure-invariant direct methods	Flack parameter: 0.01 (6)

### Fractional atomic coordinates and isotropic or equivalent isotropic displacement parameters (Å^2^)

**Table:**

	*x*	*y*	*z*	*U*_iso_*/*U*_eq_	Occ. (<1)
Cl1	0.12462 (2)	0.17275 (2)	0.49998 (5)	0.01587 (10)	
O1W	0.2500	0.2500	0.2443 (2)	0.0215 (3)	
H1	0.2178 (13)	0.2280 (11)	0.311 (3)	0.045 (7)*	
N1	0.18673 (8)	0.21731 (7)	−0.11511 (17)	0.0151 (3)	
C1	0.19934 (9)	0.15212 (8)	−0.0531 (2)	0.0166 (3)	
H1A	0.2490	0.1283	−0.0780	0.020*	
C2	0.14070 (9)	0.12028 (8)	0.0456 (2)	0.0179 (3)	
H2	0.1495	0.0744	0.0892	0.021*	
C3	0.06819 (9)	0.15569 (8)	0.0813 (2)	0.0181 (3)	
H3	0.0272	0.1345	0.1503	0.022*	
C4	0.05662 (9)	0.22220 (8)	0.0150 (2)	0.0206 (3)	
H4	0.0072	0.2468	0.0373	0.025*	
C5	0.11688 (10)	0.25259 (9)	−0.0834 (2)	0.0185 (3)	
H5	0.1093	0.2983	−0.1289	0.022*	
C6	0.2500	0.2500	−0.2232 (3)	0.0204 (5)	
H6A	0.2754	0.2141	−0.2978	0.024*	0.50
H6B	0.2246	0.2859	−0.2978	0.024*	0.50

### Atomic displacement parameters (Å^2^)

**Table:**

	*U*^11^	*U*^22^	*U*^33^	*U*^12^	*U*^13^	*U*^23^
Cl1	0.01387 (14)	0.01789 (17)	0.01585 (16)	−0.00100 (12)	0.00111 (14)	0.00074 (14)
O1W	0.0237 (8)	0.0210 (8)	0.0199 (9)	−0.0049 (6)	0.000	0.000
N1	0.0153 (6)	0.0175 (6)	0.0127 (6)	−0.0044 (5)	0.0002 (5)	−0.0016 (5)
C1	0.0141 (7)	0.0166 (7)	0.0191 (8)	−0.0001 (6)	−0.0005 (6)	−0.0034 (6)
C2	0.0184 (7)	0.0149 (8)	0.0204 (8)	−0.0012 (6)	−0.0016 (6)	0.0001 (6)
C3	0.0153 (7)	0.0222 (8)	0.0166 (8)	−0.0065 (6)	0.0019 (6)	−0.0034 (7)
C4	0.0150 (6)	0.0207 (7)	0.0261 (9)	0.0011 (6)	0.0001 (7)	−0.0066 (7)
C5	0.0183 (8)	0.0154 (8)	0.0219 (9)	−0.0013 (6)	−0.0067 (6)	−0.0025 (7)
C6	0.0199 (10)	0.0282 (12)	0.0131 (11)	−0.0111 (9)	0.000	0.000

### Geometric parameters (Å, °)

**Table:**

O1W—H1	0.85 (1)	C3—C4	1.384 (2)
N1—C5	1.348 (2)	C3—H3	0.9500
N1—C1	1.351 (2)	C4—C5	1.376 (2)
N1—C6	1.4724 (18)	C4—H4	0.9500
C1—C2	1.371 (2)	C5—H5	0.9500
C1—H1A	0.9500	C6—N1^i^	1.4724 (18)
C2—C3	1.392 (2)	C6—H6A	0.9900
C2—H2	0.9500	C6—H6B	0.9900
			
C5—N1—C1	121.60 (14)	C5—C4—C3	119.82 (14)
C5—N1—C6	119.16 (12)	C5—C4—H4	120.1
C1—N1—C6	119.22 (12)	C3—C4—H4	120.1
N1—C1—C2	120.18 (15)	N1—C5—C4	119.80 (16)
N1—C1—H1A	119.9	N1—C5—H5	120.1
C2—C1—H1A	119.9	C4—C5—H5	120.1
C1—C2—C3	119.44 (15)	N1^i^—C6—N1	110.20 (18)
C1—C2—H2	120.3	N1^i^—C6—H6A	109.6
C3—C2—H2	120.3	N1—C6—H6A	109.6
C4—C3—C2	119.15 (14)	N1^i^—C6—H6B	109.6
C4—C3—H3	120.4	N1—C6—H6B	109.6
C2—C3—H3	120.4	H6A—C6—H6B	108.1
			
C5—N1—C1—C2	−0.4 (2)	C1—N1—C5—C4	0.3 (2)
C6—N1—C1—C2	−178.98 (14)	C6—N1—C5—C4	178.89 (16)
N1—C1—C2—C3	0.0 (2)	C3—C4—C5—N1	0.2 (2)
C1—C2—C3—C4	0.5 (2)	C5—N1—C6—N1^i^	97.68 (14)
C2—C3—C4—C5	−0.6 (2)	C1—N1—C6—N1^i^	−83.73 (13)

Symmetry codes: (i) −*x*+1/2, −*y*+1/2, *z*.

### Hydrogen-bond geometry (Å, °)

**Table:**

*D*—H···*A*	*D*—H	H···*A*	*D*···*A*	*D*—H···*A*
O1w—H1···Cl1	0.85 (1)	2.37 (1)	3.216 (1)	177 (2)

## References

[bb1] Almarzoqi, B., George, A. V. & Isaacs, N. S. (1986). *Tetrahedron*, **42**, 601–607.

[bb2] Barbour, L. J. (2001). *J. Supramol. Chem.* **1**, 189–191.

[bb3] Brüdgam, I. & Hartl, H. (1986). *Acta Cryst.* C**42**, 866–868.

[bb4] Bruker (2009). *APEX2* and *SAINT* Bruker AXS Inc., Madison, Wisconsin, USA.

[bb5] Flack, H. D. (1983). *Acta Cryst.* A**39**, 876–881.

[bb6] Niu, Y. Y., Wu, B. L., Guo, X. L., Song, Y. L., Liu, X. C., Zhang, H. Y., Hou, H. W., Niu, C. Y. & Ng, S. W. (2008). *Cryst. Growth Des. ***8**, 2393–2401.

[bb7] Sheldrick, G. M. (1996). *SADABS* University of Göttingen, Germany.

[bb8] Sheldrick, G. M. (2008). *Acta Cryst.* A**64**, 112–122. 10.1107/S0108767307043930 18156677

[bb9] Westrip, S. P. (2010). *J. Appl. Cryst.* **43** Submitted.

